# Systemic Glucose-Lowering Therapies and Periodontal and Peri-Implant Outcomes in Adults With Type 2 Diabetes Mellitus: A Systematic Review

**DOI:** 10.7759/cureus.117011

**Published:** 2026-09-27

**Authors:** Abadi A Hakami, Lina A Alamri, Shahad A Alghamdi, Hala T Alanazi, Ali A Alqahtani, Waleed K Almutairi, Turki R Alzahrani, Fatma M Toonisi, Maram A Alsamti, Abdulmalik M Mossa, Amirah Y Hakami

**Affiliations:** 1 Department of Nephrology, Jazan Health Cluster, Jazan, SAU; 2 Clinical Pharmacy Program, College of Health Sciences, Taibah University, Madinah, SAU; 3 College of Medicine, Al-Baha University, Al-Baha, SAU; 4 Faculty of Medicine, Tabuk University, Tabuk, SAU; 5 College of Medicine, Majmaah University, Majmaah, SAU; 6 College of Medicine, Taif University, Taif, SAU; 7 Faculty of Dentistry, Jazan University, Jazan, SAU; 8 Faculty of Medicine, Jazan University, Jazan, SAU

**Keywords:** antidiabetic drugs, dipeptidyl peptidase-4 inhibitors, glucagon-like peptide-1 receptor agonists, glucose-lowering therapy, peri-implant bone loss, peri-implant health, periodontal disease, periodontitis, sodium-glucose cotransporter-2 inhibitors, type 2 diabetes mellitus

## Abstract

Type 2 diabetes mellitus (T2DM) and periodontitis are bidirectionally linked. Systemic glucose-lowering drug classes differ in their extra-glycaemic effects on inflammation and bone, which could plausibly modify periodontal and peri-implant health; however, this evidence has not been synthesised across drug classes. We conducted a systematic review and evidence gap map (EGM), with sodium-glucose cotransporter-2 (SGLT2) inhibitors examined as a pre-specified evidence gap. PubMed, Cochrane Central Register of Controlled Trials (CENTRAL), Web of Science, and Scopus were searched and cross-checked against the Consensus academic corpus. Eligible studies enrolled adults with T2DM, evaluated a systemic glucose-lowering drug as the exposure, and reported a periodontal or peri-implant outcome. Studies of local drug delivery and periodontal therapy aimed at improving glycaemic control were excluded from the systematic-review layer; all study designs, including preclinical studies, were charted in the EGM. Risk of bias was assessed using the Cochrane risk-of-bias 2 (RoB 2) tool and the Newcastle-Ottawa Scale. Four studies were included: one randomised controlled trial and three observational studies. These comprised a trial of a dipeptidyl peptidase-4 (DPP-4) inhibitor (sitagliptin) added to metformin, which reported improvements in probing depth, clinical attachment level, bleeding on probing, and alveolar bone density; a cohort study comparing peri-implant marginal bone loss among users of glucagon-like peptide-1 receptor agonists (GLP-1 receptor agonists), metformin, and insulin, with the least bone loss observed among glucagon-like peptide-1 receptor agonist users; a large pharmacoepidemiological cohort evaluating DPP-4 inhibitor exposure; and a cross-sectional survey of glucagon-like peptide-1 receptor agonist users. Meta-analysis was not feasible because of substantial heterogeneity. The EGM, comprising eight drug classes and six outcome domains, showed that evidence was concentrated in glucagon-like peptide-1 receptor agonists and metformin and was almost entirely preclinical; the only high-level evidence cell was represented by a single DPP-4 inhibitor randomised controlled trial, while SGLT2 inhibitors constituted a complete evidence gap across all outcome domains. Based on the four included studies, human evidence linking systemic glucose-lowering therapy with periodontal outcomes in adults with T2DM is sparse, heterogeneous, and of low certainty, with weak signals favouring incretin-based therapies. SGLT2 inhibitors remain entirely unstudied in this context despite biological plausibility. The EGM identifies specific, high-value priorities for future randomised trials and prospective cohort studies.

## Introduction and background

Periodontitis is a chronic immune-inflammatory disease affecting the tooth-supporting tissues and is approximately twice as prevalent and more severe in people with type 2 diabetes mellitus (T2DM) than in individuals without diabetes. The relationship between T2DM and periodontitis is considered bidirectional. Persistent hyperglycaemia can promote periodontal tissue destruction through mechanisms, including impaired immune responses, increased oxidative stress, accumulation of advanced glycation end products, and dysregulated inflammatory pathways. Conversely, periodontal inflammation may contribute to systemic inflammation and adversely affect glycaemic control, potentially reinforcing a cycle of metabolic and periodontal deterioration [1-4]. Given the high prevalence of both conditions and their interconnected pathophysiology, pharmacological strategies used to manage T2DM may have implications beyond glycaemic control, including potential effects on periodontal and peri-implant health.

Different classes of glucose-lowering medications have distinct biological effects that may influence periodontal tissues, bone metabolism, inflammation, and immune responses. Metformin has been associated with anti-inflammatory and potential osteogenic effects, while thiazolidinediones can modulate inflammatory pathways and bone metabolism. Dipeptidyl peptidase-4 (DPP-4) inhibitors may influence immune and tissue-remodelling processes, and glucagon-like peptide-1 receptor agonists (GLP-1RAs) have demonstrated anti-inflammatory and potentially bone-protective effects in addition to their glucose-lowering properties. Sodium-glucose cotransporter-2 (SGLT2) inhibitors may also affect inflammatory and metabolic pathways relevant to periodontal tissues [5-11]. These class-specific biological effects provide a rationale for investigating whether systemic glucose-lowering therapy is associated with differences in periodontal or peri-implant outcomes. Importantly, such effects may be particularly relevant in patients with T2DM, in whom altered glucose metabolism and chronic systemic inflammation may already increase susceptibility to periodontal disease and impaired tissue healing.

However, evidence regarding the relationship between systemic glucose-lowering therapy and periodontal or peri-implant outcomes has not been comprehensively synthesised across medication classes. Existing systematic reviews have generally examined individual drug classes, particularly GLP-1RAs, evaluated local drug delivery as an adjunct to periodontal therapy, or focused on whether periodontal treatment improves glycaemic control. Consequently, the comparative landscape of evidence across glucose-lowering drug classes and periodontal outcome domains remains unclear, and areas in which evidence is limited or absent have not been systematically characterised.

## Review

Methods

The review was conducted and reported in accordance with the Preferred Reporting Items for Systematic Reviews and Meta-Analyses (PRISMA) 2020 statement [12].

Eligibility Criteria

Eligible studies included adults (≥18 years) with T2DM who received a systemic glucose-lowering drug from the following classes: metformin, sulfonylureas, thiazolidinediones, DPP-4 inhibitors, GLP-1RAs, SGLT2 inhibitors, insulin, or alpha-glucosidase inhibitors. Comparators were placebo, no drug, or another glucose-lowering drug class. Outcomes included periodontal clinical measures, such as probing depth (PD), clinical attachment level (CAL), bleeding on probing (BOP), plaque and gingival indices, periodontitis incidence or severity, tooth loss, peri-implant marginal bone loss, and gingival crevicular fluid (GCF) or salivary biomarkers. Eligible designs were randomised or non-randomised interventional studies and analytic observational studies. Local or topical drug-delivery studies and studies evaluating periodontal therapy for glycaemic outcomes were excluded from the systematic-review layer. Animal and in vitro studies were excluded from this layer but included in the EGM.

Information Sources and Search

PubMed/MEDLINE, CENTRAL, Web of Science, and Scopus were searched from inception to June 1, 2026, without language restrictions. Searches combined terms for T2DM ("type 2 diabetes", "type 2 diabetes mellitus", T2DM) with systemic glucose-lowering therapies ("metformin", sulfonylurea*, thiazolidinedione*, "DPP-4 inhibitor*", "dipeptidyl peptidase-4 inhibitor*", GLP-1RA*, "glucagon-like peptide-1 receptor agonist*", SGLT2 inhibitor*, "sodium-glucose cotransporter-2 inhibitor*", insulin, "alpha-glucosidase inhibitor*") and periodontal/oral outcomes ("periodontitis", "periodontal disease", "periodontal", "peri-implant*", "dental implant*", "alveolar bone", gingiv*, "gingival crevicular fluid", saliva*, biomarker*). Boolean operators (AND/OR), database-specific controlled vocabulary, and free-text terms were used. Terms for local or topical drug delivery were excluded. ClinicalTrials.gov and the WHO International Clinical Trials Registry Platform (ICTRP) were additionally searched, and backward and forward citation searching of included studies and relevant reviews was performed. Complete database-specific search strategies were recorded to ensure reproducibility.

Study Selection, Data Extraction, and Risk of Bias

Records were de-duplicated and screened by two reviewers at the title/abstract and full-text stages. A Chinese-language report was translated using machine translation (DeepL/Google) and verified against its English abstract and numeric tables. Data were extracted using a piloted form covering study characteristics, design, country, sample size, T2DM ascertainment, drug class, dose and duration, comparator, outcome definitions, effect estimates and variance, and funding. Unreported information was recorded as not reported (NR), and data provenance was documented. Risk of bias was assessed using the RoB 2 tool [13] for randomised trials and the Newcastle-Ottawa Scale (NOS) for observational studies [14].

Synthesis

Because the included studies differed substantially in drug classes, comparators, study designs, follow-up periods, and outcome measures, quantitative synthesis was not performed. Findings were synthesised narratively by glucose-lowering drug class. Descriptive counts and percentages were calculated using Microsoft Excel 2026 (Microsoft® Corp., Redmond, WA).

Evidence Gap Map

An EGM was constructed to complement the human-focused systematic review and to characterise the broader preclinical evidence base. The a priori framework comprised eight systemic glucose-lowering drug classes and six oral or periodontal outcome domains, forming an 8 × 6 matrix. Randomised and non-randomised human studies, animal studies, and in vitro studies were charted, while local drug-delivery studies and studies evaluating periodontal therapy for glycaemic outcomes remained excluded.

Each cell was coded for evidence volume, represented by the number of studies, and the highest available evidence level: randomised controlled trial, human observational study, human case report, animal in vivo study, in vitro study, or no evidence. Cells were classified as containing human evidence, preclinical-only evidence, or no evidence. The coding was recorded in the EGM workbook. The EGM describes the distribution and volume of evidence but does not estimate effect sizes; therefore, a filled cell indicates the presence and volume of evidence rather than treatment efficacy or certainty.

Results

Study Selection

The searches identified 1,268 records: 208 from PubMed, 34 from CENTRAL, 590 from Web of Science, and 436 from Scopus. After removal of 189 duplicates, 1,079 records were screened by title and abstract, and 1,060 were excluded. Nineteen reports underwent full-text assessment, of which 15 were excluded because they were preclinical studies, reviews, local drug-delivery studies, evaluated an exposure other than a systemic glucose-lowering drug, included a non-T2DM population, or lacked a drug-specific periodontal or oral outcome. We included four studies: one randomised controlled trial and three observational studies. No study evaluated SGLT2 inhibitors (Figure 1).

**Figure 1:**
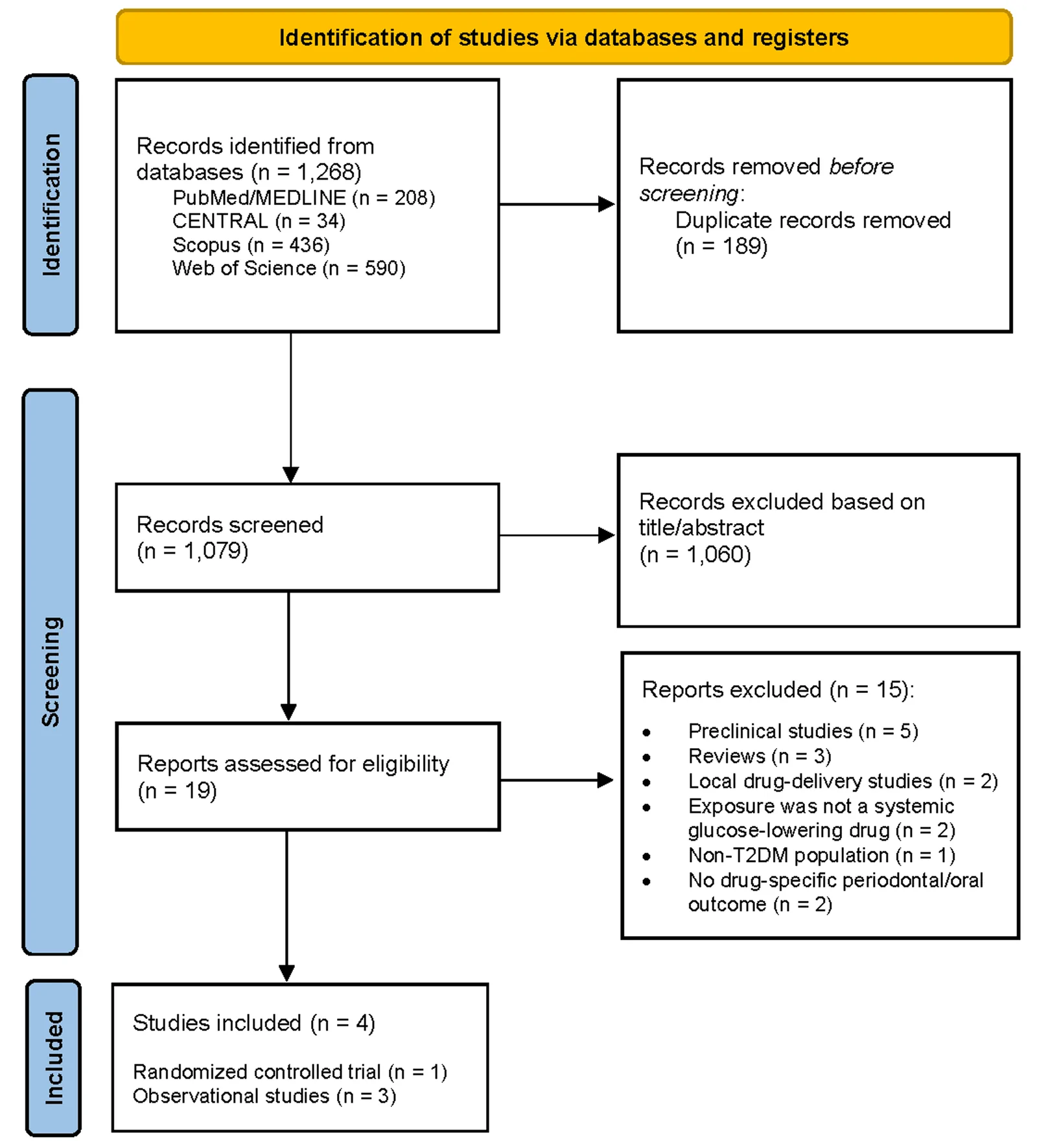
PRISMA flow diagram depicting the study selection process for the systematic review Preferred Reporting Items for Systematic Reviews and Meta-Analyses (PRISMA) flow diagram detailing the study selection process for this systematic review. Following the identification of records through database searching and other sources, duplicates were removed, and the remaining records were screened. Full-text articles were assessed for eligibility, with exclusions documented along with reasons. Studies meeting the inclusion criteria were included in the final synthesis.

Characteristics of the Included Studies

The four included studies evaluated DPP-4 inhibitors, GLP-1RAs, metformin, and insulin. The studies comprised one randomised controlled trial, one retrospective cohort study, one large pharmacoepidemiological cohort, and one cross-sectional clinical survey conducted in China and Taiwan. Study populations, exposure definitions, comparators, follow-up periods, and outcome measures varied substantially, precluding quantitative pooling (Table 1).

**Table 1:** Characteristics of the included studies Characteristics of the four included human studies evaluating associations between systemic glucose-lowering therapies and periodontal or peri-implant outcomes. The table summarises study design and sample size, glucose-lowering drug class and exposure, comparator, outcomes assessed, and the principal findings reported by each study. Study identifiers correspond to the numbered reference list. Numerical outcome data are presented as reported in the original studies; where applicable, the direction of between-group differences is indicated. Abbreviations: BMD, bone mineral density; BOP, bleeding on probing; CAL, clinical attachment level; CRP, C-reactive protein; DPP-4, dipeptidyl peptidase-4; GIP, glucose-dependent insulinotropic polypeptide; GLP-1RA, glucagon-like peptide-1 receptor agonist; HbA1c, glycated haemoglobin; NHI, National Health Insurance; PD, probing depth; T2DM, type 2 diabetes mellitus

Study; country	Design (n)	Drug class (exposure)	Comparator	Outcomes measured	Key finding
Liu [1], China	Randomised controlled trial (80)	DPP-4 inhibitor (sitagliptin) added to metformin	Metformin + basic periodontal therapy	PD, CAL, BOP, alveolar BMD, HbA1c, IL-6/CRP/TNF-α, adiponectin, leptin (3 and 6 months)	At 6 months (full-text verified): PD, 2.24 vs 2.62 mm; CAL, 2.32 vs 2.86 mm; BOP, 25.0 vs 37.5%; HbA1c, 5.07 vs 5.89% (all P = 0.01); alveolar BMD was higher (P < 0.001).
Shi [2], China	Retrospective cohort (150/308 implants)	GLP-1RA vs metformin vs insulin	Between-class	Peri-implant radiographic marginal bone loss; BOP; PD (1 and 2 years)	Marginal bone loss: GLP-1RA < insulin < metformin (P < 0.05); no significant differences in BOP or PD.
Tseng [3], Taiwan	Retrospective NHI cohort (~78,390; propensity-score matched)	DPP-4 inhibitor (sitagliptin) ever-use	Never-use	Oral cancer (primary); gingival and periodontal disease (secondary)	Longer cumulative use was associated with a favourable periodontal-disease/oral-cancer pattern.
Shen [4], China	Cross-sectional survey (150; 74 users)	GLP-1RA/GLP-1 + GIP dual agonist	Non-users	Periodontal staging and grading; agonist exposure duration	Better staging/grading with longer agonist use (T2DM status not explicit).

Evidence Gap Map

The EGM showed a markedly uneven distribution of evidence across the eight drug classes and six outcome domains. Evidence was concentrated in GLP-1RAs and metformin and was predominantly preclinical, particularly for alveolar bone regeneration and biomarker outcomes. Human evidence was limited to clinical periodontal outcomes, peri-implant outcomes, and periodontal-disease incidence. The only randomised evidence was a single DPP-4 inhibitor trial. SGLT2 inhibitors had no evidence across any outcome domain. Sulfonylureas and alpha-glucosidase inhibitors also had little or no evidence.

Of the 48 drug-class-by-outcome cells, 17 (35%) contained evidence, and 31 (65%) were empty. Human evidence was present in eight cells (17%), whereas randomised evidence was present in only one cell (2%). Entire evidence gaps were observed for SGLT2 inhibitors and sulfonylureas, as well as for tooth loss and human GCF or salivary biomarkers. Preclinical-only evidence was concentrated in the alveolar-bone/regeneration and biomarker domains, particularly for GLP-1RAs and metformin. Thus, the EGM demonstrated substantial gaps in clinical translation.

Synthesis by Drug Class

DPP-4 inhibitors: The randomised controlled trial (n = 80) found that adding sitagliptin to metformin for six months was associated with lower PD, CAL, and BOP, together with greater alveolar bone density, compared with metformin alone [1]. Glycated haemoglobin and several inflammatory and metabolic biomarkers also improved. A large pharmacoepidemiological cohort found that sitagliptin exposure was associated with a lower risk of gingival and periodontal diseases and/or oral mucosal lesions at longer cumulative exposure, although these outcomes were secondary and administratively coded [3].

GLP-1 receptor agonists: A retrospective implant cohort reported the lowest peri-implant marginal bone loss among GLP-1RA users compared with metformin and insulin users [2]. A cross-sectional clinical survey of patients with periodontitis reported better periodontal staging and grading among participants using GLP-1RAs or dual incretin receptor agonists, with greater improvement associated with longer exposure [4]. Both studies were observational and subject to potential confounding, and the survey did not establish a causal treatment effect. Overall, the GLP-1RA evidence was predominantly preclinical.

Metformin and insulin: Evidence for these agents was limited and mixed. In the retrospective implant cohort, metformin users had greater peri-implant marginal bone loss than GLP-1RA users, whereas metformin was also used as background therapy in the DPP-4 inhibitor trial, preventing isolation of its independent periodontal effect [1,2]. No eligible clinical study evaluated sulfonylureas or thiazolidinediones in a confirmed T2DM population, or alpha-glucosidase inhibitors.

SGLT2 inhibitors: No eligible human study evaluated SGLT2 inhibitors in relation to periodontal or peri-implant outcomes, and no dedicated preclinical periodontitis model was identified. Thus, SGLT2 inhibitors represented the clearest evidence gap in the review.

Risk of Bias

The randomised trial had some concerns related to incompletely described allocation concealment and blinding, short follow-up, and single-centre recruitment [1]. The observational studies had moderate-to-serious limitations, particularly potential confounding by indication and, for the cross-sectional survey, selection and measurement bias [2-4]. Overall certainty of evidence was low to very low. Meta-analysis was not feasible because no two studies shared a sufficiently comparable drug class, comparator, and outcome with extractable data (Table 2).

**Table 2:** Detailed risk-of-bias assessment of the included studies Risk of bias was assessed using RoB 2 for the randomised controlled trial, the Newcastle-Ottawa Scale (NOS) for retrospective cohort studies, and the Appraisal Tool for Cross-Sectional Studies (AXIS) for the cross-sectional survey. The table presents domain-level judgements and supporting rationales. For the NOS, a maximum of 9 stars can be awarded, with higher scores indicating better methodological quality. Abbreviations: AAP, American Academy of Periodontology; AXIS, Appraisal Tool for Cross-Sectional Studies; BOP, bleeding on probing; CAL, clinical attachment level; DPP-4, dipeptidyl peptidase-4; EFP, European Federation of Periodontology; GIP, glucose-dependent insulinotropic polypeptide; GLP-1RA, glucagon-like peptide-1 receptor agonist; NOS, Newcastle-Ottawa Scale; PD, probing depth; RCT, randomised controlled trial; RoB 2, Cochrane risk-of-bias tool for randomised trials

Study	Design	Assessment tool	Domain	Judgement	Supporting rationale
Liu [1]	Randomised controlled trial	Cochrane RoB 2	Bias arising from the randomisation process	Some concerns	Randomisation using a “random number table” was reported; however, allocation concealment was not described.
			Bias due to deviations from intended interventions	Some concerns	Blinding of participants, clinicians, and outcome assessors was not described.
			Bias due to missing outcome data	Low risk	No substantial loss to follow-up was reported during the 6-month study period.
			Bias in measurement of the outcome	Some concerns	Blinding of outcome assessors was not described; periodontal measurements may be influenced by assessor awareness.
			Bias in selection of the reported result	Some concerns	Multiple periodontal, metabolic, inflammatory, and biochemical outcomes were assessed at multiple time points; no prespecified analysis plan was identified from the available report.
			Overall risk of bias	Some concerns	Concerns primarily relate to incomplete reporting of allocation concealment, blinding, and prespecification of analyses.
Shi [2]	Retrospective cohort	Newcastle-Ottawa Scale	Representativeness of exposed cohort	★	Hospital-based cohort with defined eligibility criteria; generalisability to the wider T2DM population is limited.
			Selection of non-exposed cohort	★	Comparator groups were drawn from the same clinical population and met the same eligibility criteria.
			Ascertainment of exposure	★	Hypoglycaemic medication exposure was determined from clinical records.
			Outcome not present at start	★	Peri-implant marginal bone levels were assessed at implant placement and during follow-up.
			Comparability of cohorts	★★	Baseline characteristics were assessed and covariance adjustment was performed; however, residual confounding by indication and treatment history remains possible.
			Assessment of outcome	★	Peri-implant marginal bone loss was assessed radiographically by an independent examiner.
			Follow-up sufficiently long	★	Outcomes were assessed at 1 and 2 years after implant placement.
			Adequacy of follow-up	0	Substantial attrition occurred at 2 years (150 patients at baseline to 74 at 2 years), creating potential attrition bias.
			Overall assessment	7/9 stars; moderate risk	Main concerns were residual confounding and substantial loss to follow-up at 2 years.
Tseng [3]	Retrospective cohort	Newcastle-Ottawa Scale	Representativeness of exposed cohort	★	Population-based National Health Insurance cohort with broad population coverage.
			Selection of non-exposed cohort	★	Never-users were selected from the same underlying National Health Insurance population.
			Ascertainment of exposure	★	Sitagliptin exposure was ascertained from National Health Insurance reimbursement records.
			Outcome not present at start	★	Patients with relevant pre-existing outcomes were excluded and incident outcomes were assessed during follow-up.
			Comparability of cohorts	★★	Propensity-score matching and weighting were used to improve comparability, with multivariable Cox regression for effect estimation.
			Assessment of outcome	★	Outcomes were identified from National Health Insurance administrative records rather than self-report.
			Follow-up sufficiently long	★	The study provided longitudinal follow-up through the predefined study end date.
			Adequacy of follow-up	★	Follow-up was based on available administrative records, with no major concern regarding study-wide loss to follow-up reported.
			Overall assessment	9/9 stars; low risk	Strong population-based ascertainment, propensity-score adjustment, and administrative outcome ascertainment. Periodontal outcomes were secondary and administratively coded, limiting directness to periodontal outcomes.
Shen [4]	Cross-sectional survey	AXIS	Study aims and design	Low risk	The study had a clearly stated objective and defined clinical population.
			Sampling and representativeness	Some concerns	Single-centre, hospital-based recruitment limits representativeness of the wider population.
			Sample size	Some concerns	The clinical survey included 150 participants, including 74 exposed participants; no clear sample-size justification was identified.
			Exposure measurement	Some concerns	GLP-1RA/dual GLP-1RA-GIP agonist exposure was assessed in the clinical survey; objective prescription or dispensing verification was not clearly described.
			Outcome measurement	Low risk	Periodontal status was assessed using the 2018 AAP/EFP classification, incorporating clinical attachment loss, probing depth, and radiographic bone loss.
			Confounding	High risk	The observational cross-sectional design leaves substantial potential for residual confounding and cannot establish temporality between drug exposure and periodontal status.
			Statistical analysis	Some concerns	Cross-sectional associations between agonist exposure duration and periodontal staging/grading cannot establish causality.
			Overall risk of bias	High risk	Main concerns were the single-centre cross-sectional design, limited sample size, potential residual confounding, and inability to establish temporal or causal relationships.

Discussion

Main Findings

This systematic review identified a small and heterogeneous human evidence base examining systemic glucose-lowering therapies in relation to periodontal and peri-implant outcomes in adults with T2DM. Only four studies met the inclusion criteria, comprising one randomised controlled trial and three observational studies. The randomised trial suggested favourable periodontal and alveolar bone outcomes with sitagliptin added to metformin, while observational studies suggested potentially favourable periodontal or peri-implant findings among glucagon-like peptide-1 receptor agonist users and with longer sitagliptin exposure. However, differences in study designs, comparators, exposure definitions, and outcome measures prevented quantitative synthesis. Overall, the available evidence remains insufficient to establish a causal effect of any glucose-lowering drug class on periodontal health.

Clinical and Research Implications

The findings suggest that incretin-based therapies, particularly DPP-4 inhibitors and glucagon-like peptide-1 receptor agonists, warrant further investigation because the available studies provide preliminary signals of potentially beneficial periodontal and bone-related effects. These findings should nevertheless be interpreted cautiously because the observational studies are susceptible to residual confounding, selection bias, and limitations in outcome ascertainment. The evidence gap map further demonstrated that clinical evidence is sparse relative to the substantial preclinical literature. Of particular importance, no eligible human study evaluated SGLT2 inhibitors in relation to periodontal or peri-implant outcomes. Future studies should prioritise prospective cohorts and adequately powered randomised trials using standardised periodontal and peri-implant outcomes, clearly defined drug exposures, and sufficiently long follow-up.

Strengths and Limitations

A strength of this review was the inclusion of multiple systemic glucose-lowering drug classes and both periodontal and peri-implant outcomes, supplemented by an evidence gap map to identify areas of limited or absent research. However, the conclusions are constrained by the small number of eligible human studies and substantial clinical and methodological heterogeneity. The predominance of observational evidence limits causal inference, while the cross-sectional design of one study prevents assessment of temporality. Meta-analysis was therefore not feasible. In addition, the evidence gap map describes the distribution and volume of evidence rather than treatment efficacy or effect direction. These limitations support cautious interpretation and highlight the need for higher-quality clinical evidence.

## Conclusions

Evidence linking systemic glucose-lowering therapies with periodontal and peri-implant outcomes in adults with T2DM remains limited, heterogeneous, and predominantly observational. Although preliminary findings suggest potentially favourable periodontal effects of incretin-based therapies, the available evidence is insufficient to establish causality or support clinical recommendations. SGLT2 represent an important evidence gap. Well-designed prospective studies and randomised controlled trials using standardised periodontal and peri-implant outcomes are needed to clarify these associations.

## References

[1] Liu K, Ahemaiti A, Tuernisaguli K, Gulinuer A (2023). Effect of metformin combined with DPP-4 inhibitor on alveolar bone density in patients with type 2 diabetes mellitus and chronic periodontitis. Shanghai Kou Qiang Yi Xue.

[2] Shi S, Ding F, Liu X (2021). Clinical and radiographic variables related to implants with simultaneous grafts among type 2 diabetic patients treated with different hypoglycemic medications: a retrospective study. BMC Oral Health.

[3] Tseng CH (2017). Sitagliptin and oral cancer risk in type 2 diabetes patients. Oncotarget.

[4] Shen Y, Zhang M, Yang T (2026). The promoting roles of GLP1R and GIPR in stemness maintenance and multiple lineage-specific differentiation of PDLSCs. Cell Mol Biol Lett.

[5] Takefuji Y (2024). Oral health and diabetes updates. Br Dent J.

[6] Botelho J, Mascarenhas P, Viana J (2022). An umbrella review of the evidence linking oral health and systemic noncommunicable diseases. Nat Commun.

[7] Luo H, Wu B, Kamer AR (2022). Oral health, diabetes, and inflammation: effects of oral hygiene behaviour. Int Dent J.

[8] Kudiyirickal MG, Pappachan JM (2015). Diabetes mellitus and oral health. Endocrine.

[9] Goldenberg RM, Berard LD, Cheng AY, Gilbert JD, Verma S, Woo VC, Yale JF (2016). SGLT2 inhibitor-associated diabetic ketoacidosis: clinical review and recommendations for prevention and diagnosis. Clin Ther.

[10] Ghezzi C, Loo DD, Wright EM (2018). Physiology of renal glucose handling via SGLT1, SGLT2 and GLUT2. Diabetologia.

[11] Kolesnik E, Scherr D, Rohrer U, Benedikt M, Manninger M, Sourij H, von Lewinski D (2022). SGLT2 inhibitors and their antiarrhythmic properties. Int J Mol Sci.

[12] Page MJ, McKenzie JE, Bossuyt PM (2021). The PRISMA 2020 statement: an updated guideline for reporting systematic reviews. Rev Esp Cardiol (Engl Ed).

[13] Crocker TF, Lam N, Jordão M (2023). Risk-of-bias assessment using Cochrane's revised tool for randomized trials (RoB 2) was useful but challenging and resource-intensive: observations from a systematic review. J Clin Epidemiol.

[14] Hartling L, Milne A, Hamm MP, Vandermeer B, Ansari M, Tsertsvadze A, Dryden DM (2013). Testing the Newcastle Ottawa Scale showed low reliability between individual reviewers. J Clin Epidemiol.

